# 237. *Elizabethkingia* species: An Emerging Cause of Healthcare-Associated Bloodstream Infection Among Immunocompromised Hosts

**DOI:** 10.1093/ofid/ofad500.310

**Published:** 2023-11-27

**Authors:** Adam Stewart, Kevin Laupland, Felicity Edwards, David Paterson, Patrick N A Harris

**Affiliations:** University of Queensland, Brisbane, Queensland, Australia; Queensland University of Technology, Brisbane, Queensland, Australia; Queensland University of Technology, Brisbane, Queensland, Australia; National University of Singapore; Pathology Queensland/University of Queensland, Brisbane, Queensland, Australia

## Abstract

**Background:**

*Elizabethkingia* species are environmental opportunistic pathogens that are a rare but potentially severe cause of bloodstream infection (BSI). The objective of this study was to determine the incidence, risk factors, and outcomes of *Elizabethkingia* species BSI in a large Australian population.

**Methods:**

Retrospective, laboratory-based surveillance was conducted among residents of Queensland, Australia (population approximately 5 million) over a twenty-year study period. All microbiological data was collected from a government-funded, centralised laboratory service. Clinical and outcome information was obtained from statewide databases.

**Results:**

During 86 million person-years of surveillance, 112 incident *Elizabethkingia* species BSI occurred for an annualized age and sex-standardized incidence of 1.4 per million residents. *Elizabethkingia* species isolates were identified as *E. meningoseptica in* 104 (93%) and were not speciated in 8 (7%). Fifty-three percent were male, and the median age was 69 years (IQR, 55-80). Almost 80% were hospital onset or healthcare-associated BSI, and the median length of hospital stay being 10 days (IQR, 5-30). Comorbidity was common with a median Charlson Co-morbidity Index of 3 (IQR, 1-4), with 25% having active malignancy. Almost 80% were monomicrobial infections, with the majority having no focus identified. Ninety-day overall case-fatality rate was 23%. *In vitro* susceptibility to ciprofloxacin, sulfamethoxazole, piperacillin-tazobactam was 75%, 71%, 18%, respectively; all isolates were resistant to vancomycin.

**Conclusion:**

*Elizabethkingia* species BSI is an important cause of opportunistic infection, particularly among vulnerable hosts. Although uncommon, it is associated with a high case-fatality rate and limited antibiotic treatment options due to intrinsic resistance mechanisms.

**Disclosures:**

**David Paterson**, AMR Action Fund: Board Member|Entasis: Board Member|Merck: Board Member|Pfizer: Board Member|QPex: Board Member|Venatorx: Board Member **Patrick N A Harris, PhD, DTMH, MRCP, FRACP, FRCPA**, Merck: Advisor/Consultant|Opgen: Advisor/Consultant|Sandoz: Advisor/Consultant

